# The Prevalence of Irritable Bowel Syndrome and Its Co-existence With Migraine in Medical and Non-medical Students at Al-Baha University, Saudi Arabia

**DOI:** 10.7759/cureus.44077

**Published:** 2023-08-24

**Authors:** Ramy H Agwa, Ziyad Alharthi, Aghnar T Alzahrani, Meshari A Alghamdi, Reem A Alzahrani, Anwar A Alghamdi, Raghad H Alghamdi, Saadi R Alghamdi, Abdullah A Alghamdi

**Affiliations:** 1 Internal Medicine, Hepatology and Gastroenterology, Mansoura University, Mansoura, EGY; 2 Internal Medicine, Hepatology and Gastroenterology, Al-Baha University, Al-Baha, SAU; 3 Medicine, Al-Baha University, Al-Baha, SAU

**Keywords:** gastroenterology, medical students, prevalence, migraine, irritable bowel syndrome

## Abstract

Background: It was hypothesized that the prevalence of irritable bowel syndrome (IBS) and migraine and their co-existence are higher among medical students. In this study, we aim to establish the prevalence of IBS and migraine in the medical and non-medical students at Al-Baha University, Saudi Arabia, and to observe the association and relationship between IBS and migraine using the Rome IV diagnostic criteria for IBS and the International Classification of Headache Disorders (ICHD)-3 criteria for migraine.

Methodology: This cross-sectional study was done on the Saudi Arabian campus of Al-Baha University between July 2022 and July 2023. Al-Baha city-dwelling male and female college students aged 18 to 29 comprised the study population. A self-administered electronic questionnaire was sent online to determine the prevalence of IBS and migraine, in addition to associated risk factors. The questionnaire consisted of three sections: demographic and lifestyle data, the Rome IV criteria for diagnosing and subclassifying IBS, and the ICHD-3 criteria for diagnosing migraine.

Results: The study was conducted among 452 participants with a mean age of 21.64 years. The majority of participants were not from medical schools. The majority of medical and non-medical participants were male, at 66.6% and 63.1%, respectively. In our study, 36.9% of the individuals reported having a first-degree relative diagnosed with IBS, whereas 2.7% reported having IBS themselves. Regarding migraine, 17.9% of respondents claimed to have a first-degree relative with migraine, while 6.9% of respondents themselves reported experiencing migraine. Regarding IBS prevalence, there was no significant difference between participants from non-MBBS colleges and MBBS colleges. Similarly, there was no significant difference in migraine prevalence between these two groups (92.0% vs. 95.4%, p=0.185).

Conclusion: The current study contributes significantly to our understanding of the prevalence of IBS and migraines among medical students, as well as these individuals' demographic characteristics, familial histories, and aggravating variables.

## Introduction

Recurrent chronic gastrointestinal functional abnormalities are what is meant to be understood by the term irritable bowel syndrome (IBS). Patients who suffer from IBS frequently experience discomfort in the abdominal region, distension, and a change in bowel behavior that may manifest as constipation, diarrhea, or both [1,2]. The Rome IV criteria are now the most widely used method for diagnosing IBS without the requirement of any biochemical markers; nevertheless, to use this method, it is necessary to rule out any potential warning indications [3,4]. Although IBS is not a condition that poses a direct threat to a person's life, it has been documented that the symptoms associated with it severely impair the quality of life linked with health, including psychological status and the manifestation of concerns [5]. It is estimated that 10% to 25% of the world's population suffers from IBS [2]. In addition to this, the symptoms of this condition are among the most prevalent reasons people seek primary health care consultations [6]. Despite this, between 2.4 and 3.5 million people in the United States alone see a doctor each year because of IBS [7]. Irritable bowel syndrome has recently been found to have a prevalence of 9.2% in 53 studies that were conducted in 38 countries and comprised 395,385 people [8]. It has been estimated that between 10% and 18% of the general population in Western nations suffers from IBS [9,10]. In contrast, IBS has received little attention in countries outside of the Western world. In certain underdeveloped nations, the percentage of people who suffer from IBS ranges from 35% to 43% [11,12].

Headache has been recognized as one of the leading medical diseases that lead to global disability [13]. One of the chronic complex neuro-inflammatory disorders that can manifest in a variety of ways is migraine headache, which is a significant type of headache [14]. The condition is characterized by recurrent episodes of throbbing headache pain, which normally only affects one side of the head. Nausea and distorted vision are common side effects of the condition. There are around 1.4% of all neurological and mental illnesses that are caused by migraine headaches [14]. According to one piece of research, the expected lifetime prevalence of migraine ranged from 12% to 18% [15]. The prevalence of migraine headaches among college students is a significant public health concern. This is because of its great prevalence, the related morbidities and disabilities, and the decrease in academic performance that it causes [15]. It is common for medical students to put in long hours and be required to maintain continual concentration and study, all of which can lead to a great deal of stress and disrupted sleep patterns when subjected to high levels of stress [16].

We hypothesize that the prevalence of IBS and migraine and their co-existence are higher among medical students, and through this study, we aim to establish the prevalence of IBS and migraine among medical and non-medical students at Al-Baha University, Saudi Arabia. We also observed the association and relationship between IBS and migraines using the Rome IV diagnostic criteria and the International Classification of Headache Disorders (ICHD)-3 criteria, respectively.

## Materials and methods

This cross-sectional study was done on the Saudi Arabian campus of Al-Baha University between July 2022 and July 2023. Al-Baha city-dwelling male and female college students aged 18 to 29 comprised the study population. Participants were included using a simple random sample procedure, and inclusion and exclusion criteria were implemented to verify the validity of the study. Students were required to be enrolled at Al-Baha University and between the ages of 18 and 29, regardless of their field of study or gender, as inclusion criteria. Students who did not attend Al-Baha University, were below 18 or above 29 years of age, were in their preparatory year, had previously been diagnosed with gastrointestinal or neurological diseases other than IBS or migraine (such as peptic ulcer disease, inflammatory bowel disease, current infectious diarrhea, brain tumors, or multiple sclerosis), refused to participate in the study, or did not complete the questionnaire, were excluded.

The sample size was computed using Cochran's sample size method, and the predicted prevalence of IBS and migraine in medical and non-medical students did not reach 30%. The projected prevalence was within 5% of the actual amount, with 95% certainty. The n=(z2 Pq)/e2, where n is the sample size, z is the level of confidence, P is the expected proportion of the population with the condition, q is the complement of P, and e is the required level of precision. The z was set to 1.96 for a 95% confidence interval in this investigation, P was set to 0.3, q was set to 0.7, and e was set to 0.05. The calculated sample size was 323; an additional 30% was added to assure an appropriate response rate and account for omitted students, for a final sample size of 420.

A self-administered electronic questionnaire was sent online to determine the prevalence of IBS and migraine, in addition to the associated risk factors. The questionnaire consisted of three sections: demographic and lifestyle data, the Rome IV criteria for diagnosing and subclassifying IBS, and the ICHD-3 criteria for diagnosing migraine. The data were input into Microsoft Excel (Microsoft Corp., Redmond, WA, USA) and analyzed with SPSS Statistics version 22 (IBM Corp., Armonk, NY, USA), with categorical variables displayed as frequency and percentage and continuous variables as mean and standard deviation. The significance level was established at P less than 0.05, and an association measure was employed to determine the link between variables. The study utilized a socially acceptable technique and metrics to assure confidentiality and effective contact with the target community. The Research Ethics Committee at Al-Baha University issued approval (REC/MED/BU-FM/2022/46). All participants provided informed consent in writing.

## Results

Table 1 displays the demographics of the participants in the study. The participants' mean age was 21.64 years, with a standard deviation of 1.81 years. The majority of participants were not from medical schools, and the majority were male: 63.1% of non-medical students and 66.6% of medical students. The proportion of participants in each academic year varied, with the highest proportion in the third year (23.5%). The vast majority of participants (97.6%) were unmarried, with only a minor proportion married (2.0%) and divorced or widowed (0.4%). The majority of subjects reported not smoking (71.5%) and sleeping less than eight hours per night (59.5%). The majority of participants (68.7%) reported consuming carbonated beverages at least once each week and using coffee products at least every day (53.5%). Regarding activity levels, the majority of individuals (41.4%) indicated light exercise, followed by moderate (29.2%) and inactive (23.9%) levels, while only a few of the respondents reported extreme activity levels (5.5%).

**Table 1:** Demographic factors of the included participants

Factors	Variables	N	%
Age	Mean (standard deviation) of age	21.64 (1.81)	
Gender	Male	285	63.1%
	Female	167	36.9%
College	Non-medical colleges	301	66.6%
	Medical colleges	151	33.4%
Year	1st year	96	21.2%
	2nd year	8	1.8%
	3rd year	106	23.5%
	4th year	91	20.1%
	5th year	83	18.4%
	6th year	68	15.0%
Marital status	Single	441	97.6%
	Married	9	2.0%
	Other (divorced or widowed)	2	0.4%
Do you use tobacco products (cigarettes, shisha, e-cigarettes)?	No	323	71.5%
	Yes	129	28.5%
How many hours do you sleep?	Less than 8 hours	269	59.5%
	8 hours or more	183	40.5%
How often do you drink carbonated beverages?	I do not consume carbonated beverages	96	21.2%
	Once per week	167	36.9%
	Every other day (4 to 6 days per week)	146	32.3%
	Everyday	43	9.5%
How often do you drink coffee products?	I do not consume coffee products	70	15.5%
	Once per week	142	31.4%
	Every other day (4 to 6 days per week)	154	34.1%
	Everyday	86	19.0%
What is your exercise level?	Sedentary lifestyle	108	23.9%
	Light exercise (1 to 3 days per week)	187	41.4%
	Moderate exercise (4 to 5 days per week)	132	29.2%
	Intense exercise (6 to 7 days per week )	25	5.5%

The prevalence of IBS and migraine among research participants, as well as their familial histories, are presented in Table 2. In our study, 36.9% of the individuals reported having a first-degree relative diagnosed with IBS, whereas 2.7% reported having IBS themselves. In addition, 41.7% of individuals with IBS reported both constipation and diarrhea. Regarding migraine, 17.9% of respondents claimed to have a first-degree relative with migraine, while 6.9% of respondents themselves reported experiencing migraine.

**Table 2:** The prevalence of IBS and migraine in relation to the family history IBS: Irritable bowel syndrome

Questions	Answers	N	%
Do you have a first-degree family member who was diagnosed with IBS?	No	285	63.1%
	Yes	167	36.9%
IBS	No	440	97.3%
	Yes	12	2.7%
IBS subtype	Cannot decide	4	33.3%
	Constipation	2	16.7%
	Diarrhea	1	8.3%
	Constipation and diarrhea	5	41.7%
Do you have a first-degree family member who was diagnosed with migraine?	No	371	82.1%
	Yes	81	17.9%
Migraine	No	421	93.1%
	Yes	31	6.9%

**Figure 1:**
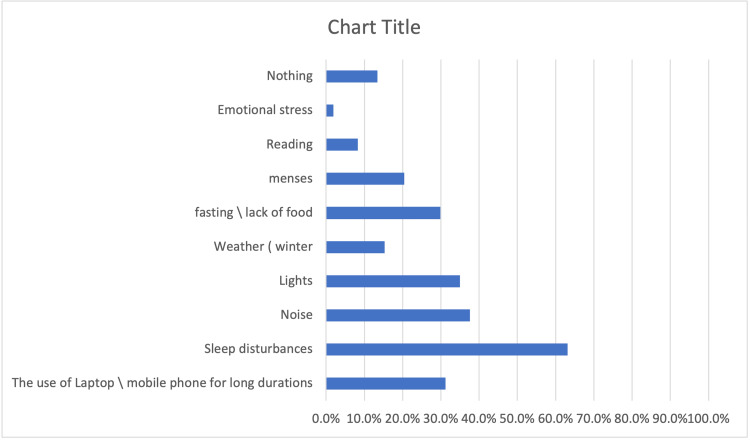
Migraine trigger factors

Figure 1 displays a summary of the aggravating factors identified by survey participants as aggravating migraine. Sleep disruptions were the most frequently stated factor, with 63.1% of people selecting them. This was followed by prolonged laptop or mobile phone use (31.2%), noise (37.6%), bright lighting (35.0%), fasting (35.0%), or lack of food (29.9%). Less often stated variables included the weather (winter), menstruation in women, reading, emotional stress, and nothing in particular.

Regarding IBS prevalence, there was no significant difference between participants from non-MBBS colleges and MBBS colleges (96.7% vs. 98.7%, p=0.213). Similarly, there was no significant difference in migraine prevalence between these two groups (92.0% vs. 95.4%, p=0.185). There was a significant gender difference in the prevalence of migraines, with a larger percentage of females reporting migraines than males (10.2% vs. 4.9%, p=0.032). Furthermore, there was a substantial difference in the prevalence of migraine between academic years, with the maximum prevalence occurring in the second year (100%) and the lowest occurring in the sixth year (2.9%) with a significant difference (p=0.000) (Table 3).

**Table 3:** The relation between prevalence of IBS and migraine with demographic factors IBS: Irritable bowel syndrome

Factors	Variables	IBS					Migraine				
		No		Yes		P-value	No		Yes		p-value
		N	%	N	%		N	%	N	%	
Age (mean (SD)		21.6 (1.7)		22.4 (2.02)		0.135	21.66 (1.81)		21.42 (1.66)		0.470
Gender	Male	277	97.2%	8	2.8%	0.793	271	95.1%	14	4.9%	0.032*
	Female	163	97.6%	4	2.4%		150	89.8%	17	10.2%	
College	Non-MBBS	291	96.7%	10	3.3%	0.213	277	92.0%	24	8.0%	0.185
	MBBS	149	98.7%	2	1.3%		144	95.4%	7	4.6%	
Year	1st year	94	97.9%	2	2.1%	0.460	94	97.9%	2	2.1%	0.000*
	2nd year	8	100.0%	0	0.0%		0	0.0%	8	100.0%	
	3rd year	105	99.1%	1	0.9%		98	92.5%	8	7.5%	
	4th year	89	97.8%	2	2.2%		84	92.3%	7	7.7%	
	5th year	80	96.4%	3	3.6%		79	95.2%	4	4.8%	
	6th year	64	94.1%	4	5.9%		66	97.1%	2	2.9%	
Marital status	Single	429	97.3%	12	2.7%	0.857	410	93.0%	31	7.0%	0.660
	Married	9	100.0%	0	0.0%		9	100.0%	0	0.0%	
	Other (divorced or widowed)	2	100.0%	0	0.0%		2	100.0%	0	0.0%	

To further study the relationship between migraine and demographic characteristics, odds ratios (ORs) can be calculated to estimate the strength of the association between these variables. The probabilities of getting migraines were 2.17 times higher in females than in males, with a p-value of 0.026 being statistically significant. There were significantly higher odds of suffering a migraine in the second year of college compared to the first (infinite odds ratio, p=0.001), and significantly decreased odds in the sixth year (OR=0.06, p=0.001). There was no significant difference between those from medical colleges and those from non-medical institutions in terms of the probabilities of having IBS or migraine, with odds ratios of 1.00 and 1.36, respectively (Table 4). 

**Table 4:** The relationship between migraine and demographic factors *Statistical significance

Variables	Odds ratio	95% CI	p-value
Gender	2.17	1.10-4.28	0.026*
Academic year			
2nd year	Infinity	N/A	0.000*
3rd year	0.11	0.01-1.04	0.055
4th year	0.47	0.06-3.71	0.473
5th year	0.28	0.03-2.34	0.273
6th year	0.06	0.01-0.34	0.003*
College type			
Non-medical	1.00	N/A	N/A
Medical	1.36	0.25-7.45	0.712

The link between the prevalence of IBS and migraine and health habits is presented in Table 5. There was no significant difference in the prevalence of IBS between smokers and non-smokers (98.% vs. 96.9%, p=0.356). Similarly, there was no significant difference in the prevalence of migraines between the two groups (7.8% vs. 6.5%, p=0.635). There was also no significant difference in the prevalence of IBS or migraine between people who slept fewer than eight hours and those who slept eight hours or more, or between participants who consumed varying quantities of carbonated beverages or coffee products. However, there was a substantial difference in IBS prevalence between activity levels. The prevalence of IBS in sedentary participants was substantially higher than in those with light, moderate, or intensive exercise routines (p=0.015). There was no significant difference between activity levels and the prevalence of migraine.

**Table 5:** IBS and migraine prevalence in relation to health habits IBS: Irritable bowel syndrome

Questions	Answers	IBS					Migraine				
		No		Yes		P-value	No		Yes		p-value
		Count	Row N%	Count	Row N%		Count	Row N%	Count	Row N%	
Do you use tobacco products (cigarettes, shisha, e-cigarettes)?	No	313	96.9%	10	3.1%	0.356	302	93.5%	21	6.5%	0.635
	Yes	127	98.4%	2	1.6%		119	92.2%	10	7.8%	
How many hours do you sleep?	Less than 8 hours	263	97.8%	6	2.2%	0.496	250	92.9%	19	7.1%	0.835
	8 hours or more	177	96.7%	6	3.3%		171	93.4%	12	6.6%	
How often do you drink carbonated beverages?	I do not consume carbonated beverages	94	97.9%	2	2.1%	0.842	86	89.6%	10	10.4%	0.469
	Once a week	163	97.6%	4	2.4%		157	94.0%	10	6.0%	
	Every other day (4 to 6 days per week)	142	97.3%	4	2.7%		138	94.5%	8	5.5%	
	Everyday	41	95.3%	2	4.7%		40	93.0%	3	7.0%	
How often do you drink coffee?	I do not consume coffee products	69	98.6%	1	1.4%	0.242	69	98.6%	1	1.4%	0.235
	Once a week	139	97.9%	3	2.1%		132	93.0%	10	7.0%	
	Every other day (4 to 6 days per week)	151	98.1%	3	1.9%		142	92.2%	12	7.8%	
	Everyday	81	94.2%	5	5.8%		78	90.7%	8	9.3%	
What is your exercise level?	Sedentary lifestyle	101	93.5%	7	6.5%	0.015	99	91.7%	9	8.3%	0.499
	Light exercise (1 to 3 days per week)	182	97.3%	5	2.7%		175	93.6%	12	6.4%	
	Moderate exercise (4 to 5 days per week)	132	100.0%	0	0.0%		122	92.4%	10	7.6%	
	Intense exercise (6 to 7 days per week)	25	100.0%	0	0.0%		25	100.0%	0	0.0%	

## Discussion

In the current study, the prevalence of IBS among medical students was found to be 2.7%, which is lower than the prevalence rates found in previous studies conducted among medical students in Asia (reported prevalence rates ranging from 12.5% to 33.3% depending on the criteria used for diagnosis [17-22]. Irritable bowel syndrome was seen in 31.7% of Ain Shams University's medical students [23]. It has the highest prevalence rate among medical students in Saudi Arabia. It was present in 38 (42.22%) out of a total of 90 medical students [24]. On the other hand, the results were different when the investigations were carried out on all students (medical and non-medical) at Damascus University, Syria [25], and Lebanon [26], since the observed IBS prevalence was substantially lower in both countries (17% and 20%, respectively). This may be because medical students have to endure longer class times, a greater number of exams, and a greater volume of reading material during their education [22]. This finding suggests that IBS is more common among medical students than was previously thought [27]. According to this finding, the incidence of IBS among medical students may not be as high as previously thought. The comparatively low prevalence of IBS in the current study may likely have been related to changes in the study population as well as the diagnostic criteria that were used in comparison to earlier investigations.

It is interesting to note that there was no significant difference detected in the prevalence of IBS between participants who attended MBBS colleges and those who attended institutions other than MBBS. The findings of this study are counter to the hypothesis that IBS would be common among medical students [28]. The fact that there is no discernible difference in prevalence between the two groups can be attributable to the fact that students attending different kinds of universities face comparable amounts of academic pressure and emotional strain; furthermore, medical colleges at Al-Baha University implement the new integrated system, which subjects the students early to clinical knowledge, and this may have altered the students' management and prevention of IBS symptoms. 

In terms of the prevalence of migraines, the current study found a prevalence rate of 6.9%, which is considerably lower than the rates that were reported in earlier studies carried out among medical students in various regions of the world, which ranged from 14% to 48% [29-31]. Based on this finding, it appears that the prevalence of migraines may differ depending on the population under research and the diagnostic criteria that are applied. The implementation of stringent diagnostic criteria for migraine, which may have resulted in the exclusion of some participants with less severe headache symptoms, may be to blame for the lower prevalence rate that was observed in the current study.

The research also discovered that there is a considerable gender difference in the prevalence of migraines, with a higher percentage of females reporting migraines than males, which is in line with findings from other studies [28,30]. This data supports the hypothesis that gender is a factor in the onset of migraines in medical students, with the possibility that this is attributable to hormonal or psychological variables.

A considerable variation in the prevalence of migraine was discovered to exist between academic years, with the highest prevalence being found in the second year of school and the lowest prevalence being found in the sixth year of school, according to the findings of the current study. These results are inconsistent with the findings reported by Galinovic et al. (2009), who found that the prevalence of migraines among medical students increased during their schooling [32]. The reasons for this increase in the prevalence of migraines among students in their second year are unknown; however, it may be related to the increasing scholastic obligations and levels of stress that students face during this year. Only a minority (eight participants) in the second year completed the questionnaire. 

The current research found that sleep disturbances were the most commonly reported trigger for migraines, followed by prolonged use of laptops or mobile phones, noise, bright lights, fasting, or a lack of food. These findings align with the findings of prior studies, which found that typical causes for migraines are either stress, a lack of sleep, or incidents that occur in daily life [28,33]. If these aggravating factors can be identified, medical students may have a higher chance of better managing their migraines by avoiding or minimizing their exposure to these triggers.

The current study found no significant difference in the prevalence of IBS or migraines between smokers and non-smokers. However, there was a significant difference in the prevalence of IBS between activity levels, with the prevalence of IBS being much greater in people who were sedentary in comparison to those who had mild, moderate, or intense exercise routines. These findings are in line with the findings of prior research, which found that a high-stress level and a lack of physical activity are risk factors for IBS [7,34]. By recognizing these potential triggers, medical students may be able to better control their IBS and migraines by adopting healthy living choices.

Strengths and limitations

Although the study employed established diagnostic criteria for both IBS and migraine, obtained IRB approval, and ensured that all participants provided informed consent, it has several limitations. As the research was done at a single university in Saudi Arabia, the applicability of the findings cannot be generalized to other countries or people. Self-reported data may be susceptible to recollection bias and social desirability bias. In addition, the study did not gather information on potential confounding variables, such as family history or medication usage, which may have hindered the capacity to discover significant links between IBS and migraine.

## Conclusions

In conclusion, the current study contributes significantly to our understanding of IBS in medical and non-medical students. We found that there are no significant differences in prevalence between medical and non-medical participants, which contradicts our hypothesis. Stress alone in medical school is not a risk factor for the development of IBS or migraine, and other factors play a role. In addition, the study shows the significance of lifestyle factors such as exercise in the prevention and treatment of IBS and migraines among medical students. Additional research is required to investigate the underlying processes of IBS and migraines in this population, as well as the efficacy of lifestyle treatments in reducing the prevalence of these disorders and the severity of their symptoms.
